# Estrogen Mediates an Atherosclerotic-Protective Action *via* Estrogen Receptor Alpha/SREBP-1 Signaling

**DOI:** 10.3389/fcvm.2022.895916

**Published:** 2022-07-05

**Authors:** Fei Xie, Xiandong Li, Yue Xu, Dongliang Cheng, Xianru Xia, Xi Lv, Guolin Yuan, Chunyan Peng

**Affiliations:** ^1^Department of Laboratory Medicine, Taihe Hospital, Hubei University of Medicine, Shiyan, China; ^2^Department of Outpatient, Taihe Hospital, Hubei University of Medicine, Shiyan, China; ^3^Department of Cardiothoracic Surgery, Taihe Hospital, Hubei University of Medicine, Shiyan, China; ^4^Hubei Key Laboratory of Embryonic Stem Cell Research, Hubei University of Medicine, Shiyan, China

**Keywords:** estrogen, estrogen receptor α, SREBP-1, atherosclerosis, dyslipidemia

## Abstract

Menopause is associated with dyslipidemia and an increased risk of cardiovascular disease, the underlying mechanism of dyslipidemia is attributed to an insufficiency of estrogen. In this study, we find that estrogen mediates an atherosclerotic-protective action *via* estrogen receptor alpha/SREBP-1 signaling. Increased lipid accumulation and low-density lipoprotein (LDL)-uptake in HepG2 cells and THP-1 macrophages were induced by treatment of mixed hyperlipidemic serum from postmenopausal women; 17β-estradiol [estrogen (E2)] (10 nM) administration significantly improved hyperlipidemic profiles, relieved fatty-liver damage and attenuated the plaque area in the heart chamber of high-fat diet (HFD)-fed ovariectomized (OVX) Apo*E*^–/^*^–^* mice. Expression of sterol regulatory element-binding protein (SREBP)-1 mRNA of circulating leukocytes in postmenopausal women was strongly correlated to the serum E2 level. Exploration of data from the Gene Expression Profiling Interactive Analysis (GEPIA) database revealed that expression of SREBP-1 protein correlated to expression of estrogen receptor (ESR)α protein in the liver, blood and in normal tissue. Genetic overexpression/inhibition of ESRα resulted in increased/decreased SREBP-1 expression as well as attenuated/deteriorated lipid deposition *in vitro*. An inhibitor of the protein kinase B/mammalian target of rapamycin (AKT/mTOR) pathway, AZD8055, abolished ESRα-induced SREBP-1 expression in HepG2 cells. Moreover, E2 and statin co-treatment significantly reduced lipid accumulation *in vitro* and hindered the progression of atherosclerosis and fatty-liver damage in OVX Apo*E*^–/^*^–^* mice. Collectively, our results suggest that estrogen could exerted its atherosclerotic-protective action *via* ESRα/SREBP-1 signaling. E2 might enhance the cellular sensitivity of statins and could be used as a novel therapeutic strategy against atherosclerotic disorders in postmenopausal women.

## Introduction

The menopause is an age-related loss of ovarian functions and a decline in gonadal steroids. In premenopausal women, 17β-estradiol (E2) is the predominant endogenous estrogen primarily and variably synthesized in the ovaries during the menstrual cycle. Depletion of ovarian follicles in the perimenopausal period leads to a steady decline in E2 production. Compared with premenopausal women, postmenopausal women are more prone to obesity and carry a higher risk of atherosclerotic cardiovascular disease (ASCVD) and have an atherogenic plasma lipid profile (1, 2). Specifically, they have greater circulating concentrations of total cholesterol (TC), low-density lipoprotein-cholesterol (LDL-C), production of very low-density lipoprotein (VLDL) triglyceride, and greater ability to remove VLDL triglyceride (2). Levels of the independent predictors of cardiovascular disease risk, apolipoprotein (Apo)B, ApoA-I and ApoAII, are also increased during menopause (3–5). These differences in concentrations and subclass distributions of lipids are likely to account (at least in part) for the cardioprotective effect of E2, which was reported be esterified via Lecithin Cholesterol Acyl Transferase (LCAT) function. E2 esters can be carried by HDL particles and could be physiologically relevant with the pathogenesis of atherosclerosis (6).

The effects of menopause are also supported by ovariectomy models in animals. They show that a depletion in circulating E2 levels results in enhanced hepatic lipid accumulation, attenuated inhibition of lipogenesis and inflammation of adipose tissue (7, 8), and these effects can be reversed by exogenous administration of E2 (9, 10). Understanding E2-caused differences in lipid metabolism and the factors involved in the regulation of lipid kinetics and plasma lipid profile (a major risk factor for cardiovascular disease) is important because there are significant differences in the ways premenopausal and postmenopausal women experience ASCVD. For decades, hormone replacement therapy has been used for the relief of menopausal symptoms for women. Conventional E2 therapy might beneficially affect adiposity and the risk of diabetes mellitus but also a perceived increased risk of breast cancer, as well as an established risk of stroke and venous thromboembolism.

The effects of E2 are mediated by the two nuclear estrogen receptors (ESRs): α and β (11). E2 activates ESRs in target cells, which act as transcription factors to regulate expression of target genes, ultimately controlling cell growth, cell differentiation and homeostasis (12). The liver impacts lipid metabolism in response to E2 signaling. More than 1000 genes display a sex bias in their expression in the liver (13) and the top biological pathways are in lipid homeostasis related to ASCVD (14). Studies using chromatin immunoprecipitation assays have revealed 43 lipid genes to be regulated by ESRα (15, 16). However, the relative contributions of E2 signaling through ESRs with regard to lipid metabolism, lipoprotein metabolism and ASCVD were not well defined.

Membrane-bound, basic helix-loop-helix leucine zipper transcription factors are referred to as “sterol regulatory element-binding proteins” (SREBPs). The latter have a central role in regulating the genes that are important for the biosynthesis and uptake of lipids (17, 18). Previously, we found that SREBP-1 expression in circulating leukocytes was downregulated significantly in patients with ASCVD (19). After re-stratification and consultation with participants after that study, we discovered that the decrease in SREBP-1 expression showed an age-related, menopause stage-dependent pattern.

Here, we evaluated the lipid profiles and expression of gonadal hormones and SREBPs in premenopausal, perimenopausal and postmenopausal women. Furthermore, to investigated if E2 signaling regulates lipid metabolism and is protective against the early stages of atherosclerosis, lipid metabolism and arterial plaques were also evaluated in a foam-cell model and in ovariectomized (OVX) ApoE^–/–^ mice. We demonstrated that E2/ESR ligands reduced LDL uptake and lipid accumulation in hepatocytes and foam cells *in vitro* through a novel non-classical E2/ESR/SREBP-1 signaling pathway, and that E2 treatment enhanced cellular statin sensitivity by perturbing cholesterol metabolism in OVX ApoE^–/–^ mice.

## Materials and Methods

### Reagents and Cell Culture

Cell-culture media [Dulbecco’s modified Eagle’s medium (DMEM), RPMI-1640, phenol red-free 1640] and fetal bovine serum (FBS) were purchased from Gibco (Grand Island, NY, United States). Inhibitor of mammalian target of rapamycin (mTOR), AZD8055, were obtained from Selleck Chemicals (Houston, TX, United States). E2 (S1709), rosuvastatin (PHR1928) and dimethyl sulfoxide (D8412) were purchased from Sigma–Aldrich (Saint Louis, MO, United States).

Antibodies against SREBP-1 (ab28481), SREBP-2 (ab30682), ESRα (ab32063), ESRβ (ab3578), GAPDH, and secondary antibodies were purchased from Abcam (Cambridge, United Kingdom). Anti-mTOR, and Anti-(p)-mTOR were purchased from Cell Signaling Technology (Danvers, MA, United States).

The human hepatic carcinoma cell line HepG2 and monocytic THP-1 were obtained from Shanghai Institute of Cell Bank (Shanghai, China). Human monocytic (THP-1) cells were cultured in phenol red-free medium supplemented with 10% FBS and incubated with 50 ng/mL of phorbol esters (PMA) for 48 h for differentiation into macrophages, THP-1 macrophages and HepG2 cells were maintained in tissue culture dishes (diameter 100 mm) in phenol red-free Dulbecco’s Modified Eagle Medium (Fisher Scientific, Waltham, MA, United States) that was supplemented with 10% (v/v) heat-inactivated and charcoal-stripped FBS (Fisher Scientific), 1% antibiotics of 50 U/mL penicillin, and 50 μg/mL streptomycin (Invitrogen, Grand Island, NY, United States) at 37°C and 5% CO2. When the initial cells (1 × 10^5^/mL) became ∼70% confluent, the cells were starved with medium low in serum (0.1% v/v FBS) for 24 h before treatments. HepG2 Cells and THP-1 macrophages were cultured with 10 nM E2 or its vehicle in the presence of 10% hyperlipidemic serum (HS) for 24 h for further staining and LDL-uptake experiments.

### Participants

A total of 231 healthy women (21–70 years) were recruited in Taihe Hospital from August 2018 to April 2020. After an overnight fast, participants provided a blood sample, underwent medical examinations and were instructed to complete a self-reported questionnaire which included detailed questions regarding menopausal status. We enrolled women who experienced natural menstrual states. The inclusion criteria were identical to those described previously (20) (Supplementary Data). Determination of menopausal status was based on the responses to questions regarding menstrual irregularity and amenorrhea in the self-reported questionnaire. “Perimenopause” was defined as the presence of menses within the previous 3 months with a reduction in cycle predictability in the year preceding examination, or 3–11 months of amenorrhea. All blood biochemical parameters were detected by standard methods, which were undertaken in the core laboratory of Taihe Hospital.

The study protocol was approved by the Medical Ethics Committee of Taihe Hospital of the Hubei University of Medicine (Hubei, China). Our study was undertaken in accordance with the ethical guidelines of Declaration of Helsinki 1964 and its later amendments.

### Preparation of Mixed Hyperlipidemic Serum

We enrolled 26 postmenopausal study participants with hypercholesterolemia. Blood samples were obtained from these women. Serum samples were mixed together and passed through a 22-nm filter (Millipore). The final concentration of biochemicals and hormones was measured and is illustrated in Supplementary Table S1. “hypercholesterolemia” was defined as TC ≥ 6.2 mmol/L and/or LDL-C ≥ 4.1 mmol/L (21).

### Transfection of ESRα, SREBP-1

Transient transfection was performed with Lipofectamine 3000 (Lipo3000; Invitrogen, United States) transfection kits. HepG2 cells were cultured with 5% FBS and 10 nM E2 and transfected with ESRα- cloned vector (ESRα Overexpression), controlled vector (pcDNA), and short-hairpin RNAs (shRNAs) according to the manufacturer’s instructions. All transfection reagents were synthesized by VectorBuilder Inc. (Guangzhou, China), The specific shRNA sequences used are listed in the Supplementary Table S3. After 48 h of transfection with lipo3000, transfection effectiveness was evaluated in qRT-PCR and Western Blots.

### qPCR and Western Blotting

Total RNA was extracted from cells using TRIzol^®^ Reagent (Invitrogen, CA, United States) according to manufacturer instructions. mRNA expression was quantified by real-time reverse transcription-quantitative polymerase chain reaction (RT-qPCR), ChamQ SYBR qPCR Master Mix (Nanjing Vazyme Biotech Co., Ltd., Nanjing, China), Glyceraldehyde-3-phosphate dehydrogenase (*GAPDH*) was regarded as an endogenous reference. PCR were performed by using Bio-Rad CFX Manger system (Bio-Rad Laboratories, Hercules, CA, United States). Relative gene expression was normalized to that of the housekeeping gene GAPDH and calculated using the formula 2^–ΔΔCT^. The sequences of gene-specific primers are listed in Supplementary Table S2.

To determine the expression of SREBP-1, SREBP-2, ESRα, mTOR, and p-mTOR in THP-1 and HepG2 under different treatment were detected by western blot. The cells were lysed in the cold lysis buffer and the proteins were separated on SDS-PAGE and immunoblotted with indicated antibodies as described previously (22). An enhanced chemiluminescence (ECL) detection system was used to detect immunocomplexes and images were acquired using the ChemiDoc™ Touch imaging system (Bio-Rad Laboratories, Hercules, CA, United States). Detailed qPCR and WB methods were described in Supplementary Method.

### Oil Red O Staining

HepG2 Cells and THP-1 macrophages were pretreated with 10 nM E2 or its vehicle in the presence of 10% HS for 24 h. The cells were washed with PBS and then applied to Oil Red O (ORO) staining assay according to the protocol of a commercial kit (Abcam, #133102). Briefly, cells were first washed with PBS and fixed with formalin solution for 15 min. The fixed lipid droplets were then stained with Oil Red O solution for 30 min at room temperature. After washed with PBS for third times, microscope images were taken to visualize red oil droplets staining in differentiated cells (Leica, Wetzlar, Germany). ImageJ (National Institutes of Health) was used to measure the mean staining area intensity (23).

### Low-Density Lipoprotein-Cholesterol Uptake Assay

Low-density lipoprotein-cholesterol uptake was determined using an LDL Uptake Assay Kit according to manufacturer (133127; Abcam) instructions as described previously (22) and detailed methods were described in Supplementary Method S1.4.

### Immunofluorescence Staining of Cells

Cells were seeded into six-well plates. The next day, cells underwent different treatments for 24 h followed by fluorescence staining according to standard methods as described previously. Immunofluorescence images were visualized and photographed using a laser scanning confocal microscope (FV3000RS; Olympus, Tokyo, Japan). ImageJ (National Institutes of Health) was used to measure the mean fluorescence intensity (23) (Supplementary Method S1.5).

### Surgical Model of Menopause and Atherogenesis in Mice

Female ApoE^–/–^ mice (5 weeks) were purchased from Biocytogen (Nanjing, China). Mice were maintained in a temperature-controlled room and exposed to a 12-h light–dark cycle. After 3 weeks of acclimatization, mice were randomized to undergo bilateral ovariectomy after the induction of anesthesia (1% pentobarbitone). Seven days after surgery, mice were stratified into three groups of five in the Part 1 and stratified into four groups of eight in Part 2. E2 and Rosuvastatin were dissolved in physiologic saline and administered into mice by intragastric administration. The diagrams of animal research in part 1 and part 2 were shown in the Supplementary Method S1.6. Animal experiments followed the *Guide for the Care and Use of Laboratory Animals* (National Institutes of Health, Bethesda, MD, United States) and their conduct was approved by the Animal Care and Use Committee of Hubei University of Medicine.

### Assessment of Aortic Atherosclerotic Lesions

After the treatment, mice were anesthetized with Pentobarbital sodium salt (1% pentobarbitone). The liver, heart, and arterial tree were dissected carefully and were incubated with physiologic saline and fixed in 4% formalin, then dehydrated with sucrose gradient. Tissues were embedded in Tissue Freezing Medium and serially sectioned at 8 μm using a Thermo cryostat (Thermo Scientific, Waltham, MA, United States). To quantify the dimensions of atherosclerotic lesions, 10–12 sections were stained with ORO and counterstained with hematoxylin. Images were captured with Leica CS2 and lesion areas were quantified with ImageJ (23).

### Statistical Analyses

The continuous variables are expressed as mean ± standard deviation (SD) or as median (inter-quartile range), as appropriate, based on distributions. Continuous variables were compared by the student’s *t*-test or Mann–Whitney *U* test. Correlation analysis between blood lipids levels and E2 levels was performed using the non-parametric Spearman’s rank correlation test. Student’s *t*-test was used to evaluate the significance of pairwise in differences. All statistical significance levels were set at *p* < 0.05 (two-sided) and all statistical analyses were performed using SPSS version 23.0 (SPSS Inc., Chicago, IL, United States).

## Results

### The Serum E2 Level Is Negatively Correlated With Levels of Total Cholesterol and Low-Density Lipoprotein-Cholesterol in Postmenopausal Women

**FIGURE 1:**
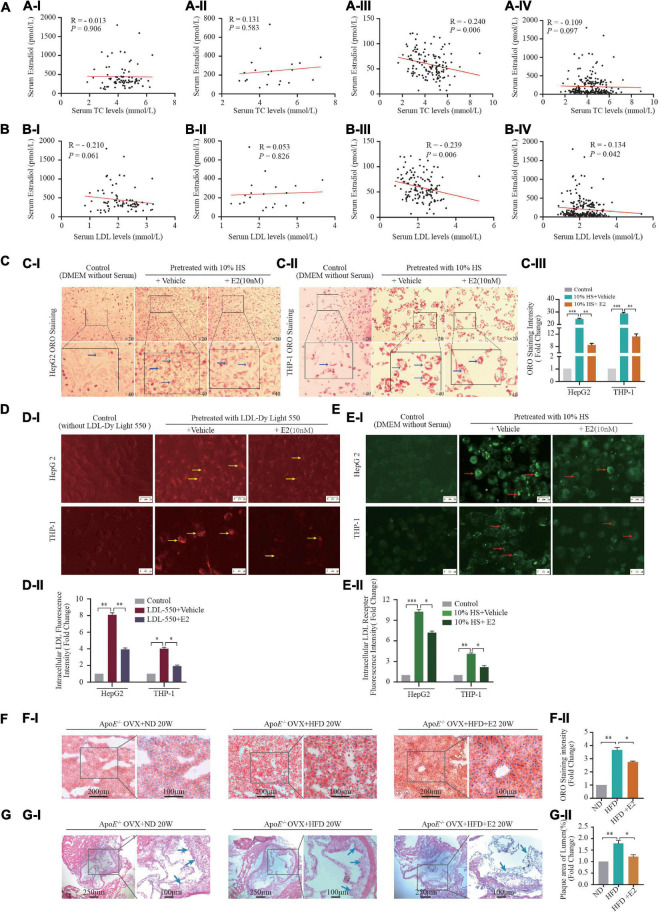
Estrogen affects lipid metabolism *in vivo* and *in vitro*. **(A,B)** A total of 231 healthy women (21–70 years) who experienced natural menstrual states were recruited. Correlation of serum 17-β estradiol [estrogen (E2)] levels and TC in premenopausal **(AI)** (*n* = 80), perimenopausal **(AII)** (*n* = 20), postmenopausal **(AIII)** women (*n* = 131), and all female participants **(AIV)** (*n* = 231); correlation of serum E2 levels and LDL-C in premenopausal **(BI)** (*n* = 80), perimenopausal **(BII)** (*n* = 20), postmenopausal **(BIII)** (*n* = 131) women and all female participants **(BIV)** (*n* = 231); **(C)** In order to build an *in vitro* foam-cell environment to mimic the postmenopausal state, mixed hyperlipidemic serum (HS) from post-menopause females (*n* = 26) were used to pretreat HepG2 cells and THP-1 macrophages. After stimulated by 5% FBS and 10% HS with or without E2 treatment for 24 h, intracellular lipid accumulation *in vitro* was measured by the ORO staining assay. Representative images from at least three independent experiments are presented **(CI)**. Total area of lipid droplets normalized to cell number (*n* = 5∼8 cells/field/slice, *n* = 3 slices/group) was determined using Image J software and shown in **(CII)**. **(D)** HepG2 cells and THP-1 macrophages stimulated by fluorescence labeling LDL (LDL-Dy Light 550) were treated with vehicle or with E2 (10 nM) for 24 h, Representative fluorescence images of intracellular LDL in HepG2 cells and THP-1 macrophages were shown in **(DI)**. Unstimulated HepG2 cells and THP-1 macrophages were set as controls. Total area of labeling LDL normalized to cell number (*n* = 10 cells/field/slice, *n* = 3 slices/group) was determined using Image J software. Representative images from at least three independent experiments are presented **(DII)**. Red light indicates labeling LDL. **(E)** After labeling LDL visualization **(CI)**, cells were blockade with Cell-Based Assay Blocking Solution for 30 min and stained with rabbit anti-LDL receptor primary antibody and DyLight 488-conjugated secondary antibody. The cells were then subjected to microscopic analysis for detection of LDLR expression, which are indicated by green light. Total area of LDLR expression normalized to cell number (n = 10 cells/field/slice, n = 3 slices/group) was determined using Image J software and shown in **(EII)**. **(F)** Eight-week-old ApoE^–/–^ mice were subjected to ovariectomy. Seven days later, they were randomized to three groups: normal diet (NF) group (*n* = 5), high-fat diet (HFD) group (*n* = 5), or HFD + E2 (1 μg/kg/day) administration group (*n* = 5). Treatments continued for 20 weeks until euthanasia. After euthanasia, liver tissue were prepared as rapid freezing section and subjected to ORO staining for detection of lipids accumulation. Representative images of liver ORO staining are shown **(FI)** (*n* = 3 sections/mouse/group, *n* = 5 mice/group). Densitometric quantification was conducted using Image J and values are expressed as the fold change compared with control and are presented as mean ± SEM (*n* = 5 mice/group). **(G)** Heart tissue were prepared as rapid freezing section and subjected to H&E staining for detection of plaque area of the three groups of mice (*n* = 3 sections/mouse/group, *n* = 5 mice/group). Densitometric quantification was conducted using Image J and values are expressed as the fold change compared with control and are presented as mean ± SEM (*n* = 5 mice/group). Student’s *t*-test was used to evaluate the significance in differences between two groups of observations, *P < 0.05; **P < 0.01; ***P < 0.001. E2, 17β-estradiol; HS, hyperlipidemic serum from postmenopausal women; LDL, low-density lipoprotein; LDLR, LDL receptor; OVX, ovariectomized; TC, total cholesterol; TG, triglycerides; HDL-C, high-density lipoprotein-cholesterol; LDL-C, low-density lipoprotein-cholesterol; ORO, Oil red O; OVX, ovariectomy.

The basic clinical characteristics of 231 women (80 premenopausal, 20 perimenopausal, 131 postmenopausal) are shown in Supplementary Table S4. As shown in Figure 1, serum E2 levels were negatively correlated with TC (*r* = −0.240, *P* = 0.006) (Figure 1AIII) and LDL-C (*r* = –0.239, *P* = 0.006) levels (Figure 1BIII) in postmenopausal women. Moreover, the serum level of E2 was negatively correlated with the LDL-C level (*r* = –0.134, *P* = 0.042) (Figure 1BIV) in the total study cohort.

### Effects of E2 on Hyperlipidemic Serum-Treated Cells and Ovariectomized ApoE^–/–^ Mice

In postmenopausal females, circulating levels of hormones change dramatically because ovarian function begins to decline; E2 secretion decreases gradually and the homeostasis of lipid kinetics is disrupted (24). We wished to build an *in vitro* foam-cell environment to mimic the postmenopausal state. Hence, we used mixed HS from postmenopausal women to treat THP-1 cells. As shown in Figures 1C–E, 10% HS treatment increased intracellular lipid deposition (*P* < 0.001), LDL-uptake (*P* < 0.001), LDL-receptor expression (*P* < 0.001) in HepG2 cells and THP-1 macrophages, compared with those in the control groups. Furthermore, these effects were attenuated by treatment with E2 (10 nM).

We used HFD-fed ApoE^–/–^ mice that had undergone ovariectomy as a comorbidity model of postmenopausal status and atherosclerosis in women on the basis that E2 deprivation by OVX and HFD consumption in ApoE^–/–^ mice induces dyslipidemia and atherosclerosis. HFD consumption increased the bodyweight (Supplementary Figure S1), serum lipid level (Supplementary Figure S2), hepatic lipid accumulation (Figure 1F) and plaque area in heart chambers (Figure 1G) in OVX mice compared with that in OVX mice fed a normal-fat diet. Administration of E2 (10 nM) had no effect on bodyweight or serum levels of LDL-C, TG, or TC (Supplementary Figure S1). However, ORO staining in the liver and hematoxylin and eosin (H&E) staining in the heart revealed that E2 (10 nM) attenuated the effect of HFD induced-fatty liver damage and atherosclerotic-plaque formation in OVX mice (Figures 1F,G).

### SREBP-1 Mediates the Effect of E2 on Foam-Cell Formation

**FIGURE 2:**
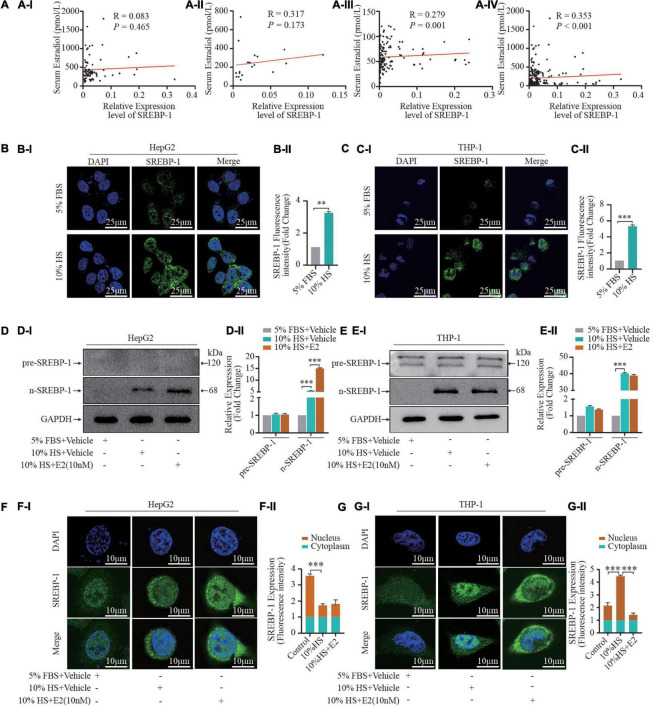
Estrogen influences SREBP-1 expression. **(A)** A total of 231 healthy women (21–70 years) who experienced natural menstrual states were recruited. The correlation of serum estrogen levels and SREBP-1 mRNA expression in leukocytes in premenopausal **(AI)** (*n* = 80), perimenopausal **(AII)** (*n* = 20), postmenopausal **(AIII)** (*n* = 131) women, and all female participants **(AIV)** (*n* = 231). **(B,C)** HepG2 and THP-1 macrophages stimulated by 5% FBS and 10% HS for 24 h, SREBP-1 immunofluorescence were visualized under confocal microscopy; DAPI stains the nucleus (blue), SREBP-1 (green) **(BI,CI)**. Densitometric quantification was conducted using Image J and values are expressed as the fold change compared with control and are presented as mean ± SEM **(BII,CII)** (*n* = 5∼8 cells/field/slice, *n* = 3 slices/group). **(D,E)** Pre-SREBP-1 and n-SREBP-1 were further analyzed by western blot. Densitometric quantification was conducted using Image J and values are expressed as the fold change compared with control and are presented as mean ± SEM **(DII,EII)**. **(F,G)** HepG2 and THP-1 cells pretreated with or without 10% HS for 24 h in the presence or absence of E2 (10 nM). Expression and location of SREBP-1 and nuclear SREBP-1 detected by immunofluorescence **(FI,GI)** (*n* = 1 cells/field, *n* = 5∼8 fields/slice, *n* = 3 slices/group), the relative expression intensity in nucleus vs in cytoplasm were illustrated in **(FII,GII)**. Data illustrated on the bar graph are the mean ± SD. All representative images presented were repeated in three independent experiments. Student’s *t*-test was used to evaluate the significance in differences between two groups of observations, *P < 0.05; **P < 0.01; ***P < 0.001.

Accumulating evidence suggests that reprogramming of fatty-acid metabolism modulates lipid disorders in cardiovascular disease. To investigate if E2 influenced lipogenesis during foam-cell formation, we examined the effects of HS on expression of SREBP-1 (the master transcriptional regulator of fatty-acid biosynthesis). First, we examined the correlation between serum E2 levels and SREBP-1 mRNA expression in leukocytes in female participants with different physiological status (Figure 2A). Expression of SREBP-1 mRNA was positively correlated with the E2 level in postmenopausal women (*R* = 0.279, *P* = 0.001) as well as in all female participants (*R* = 0.353, *P* < 0.001). Second, we used different concentrations of HS to treat THP-1 cells. HS pretreatment for 24 h increased expression of the cleaved form of SREBP-1 (n-SREBP-1) (Supplementary Figure S2). Interestingly, expression of the pre-form of SREBP-1 (pre-SREBP-1) was not affected by pretreatment with HS, which suggest that selective SREBPs signaling was involved in foam-cell formation. Notably, treatment with 10% HS led to maximal foam-cell formation and SREBP-1 expression (Supplementary Figure S2).

Furthermore, given that n-SREBP-1 is the mature form of SREBP, which can regulate gene expression, we next investigated the effect of E2 on n-SREBP-1 expression. We found that E2 promoted n-SREBP-1 expression which was induced by HS pretreatment in HepG2 and THP-1 macrophages (Figures 2B–E). Moreover, we examined the cellular localization of SREBP-1 by immunofluorescence assay before and after HS treatment (Figures 2F,G). At baseline, most SREBP-1 was localized to the nucleus in HePG2 cells while its immunoreactivity was very weak in THP-1 cells. Upon addition of 10% HS, we observed a significant increase of immunoreactivity in HePG2 cells and THP-1 cells; most SREBP-1 was localized to the cytoplasm in HePG2 cells while most SREBP-1 was localized to the nucleus in THP-1 cells under HS treatment. Furthermore, supplementation with E2 enhanced the overall intracellular expression of SREBP-1 (Figures 2F,G). Interestingly, variation in the nucleus-to-cytoplasmic immunoreactivity (NC ratio) of SREBP-1 which was induced by E2 in the two cell types was not consistent (Figures 2F,G). The NC ratio of SREBP-1 was decreased in HepG2 cells upon E2 treatment, but the NC ratio was increased in THP-1 cells upon E2 treatment. These findings uncovered the dynamically integrated and finely tuned lipid-metabolism signaling of E2.

### E2 Influenced ESRα Expression

ESRα is a nuclear hormone receptor that governs expression of the target genes involved in numerous metabolic pathways, including those guiding mitochondrial oxidative phosphorylation and lipid metabolism (25). Postmenopausal women had reduced E2 levels and decreased ESRα expression, so we speculated that E2 supplementation may restore expression of ESRs and potentially mediate the regulatory effects of E2. Treatment with 10% HS enhanced the immunoreactivity of ESRα in THP-1 cells and HepG2 cells, in particular the mean fluorescence intensity in the nucleus was increased dramatically (Supplementary Figure S3), which implied that E2 transcription activated ESRα. Furthermore, E2 treatment increased expression of ESRα protein in HepG2 cells and decreased expression of ESRα protein slightly in THP-1 cells.

### SREBP-1 Mediates the Lipid Regulatory Effect of E2 *via* ESRα

**FIGURE 3:**
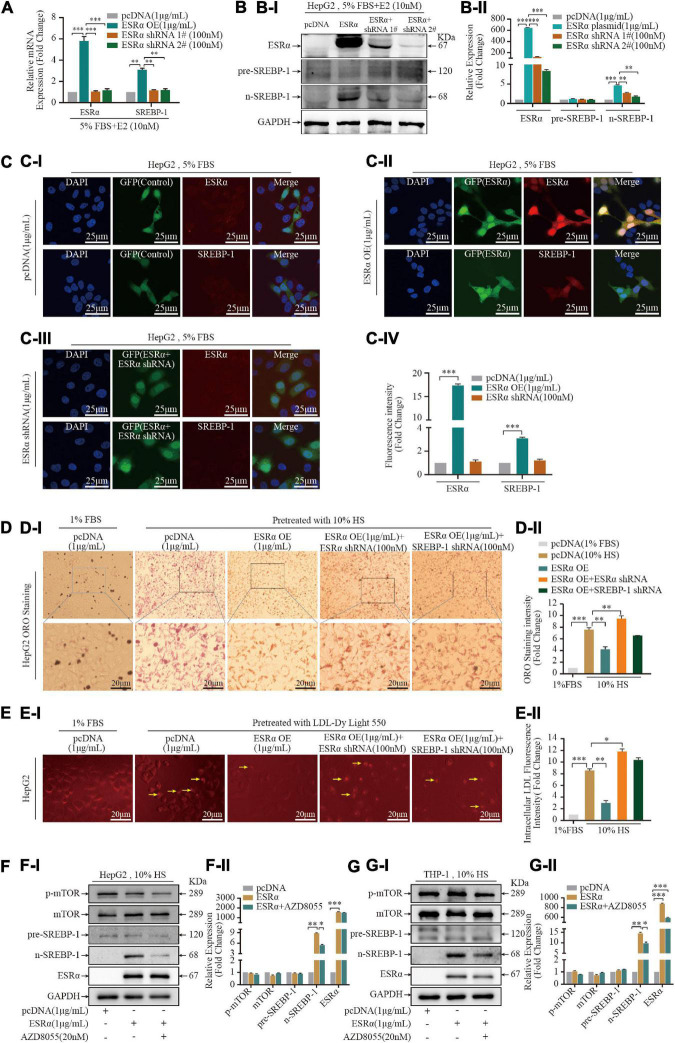
Estrogen enhanced statin sensitivity both *in vivo* and *in vitro*. **(A)** HepG2 cells and THP-1 macrophages stimulated by 5% FBS and 10% HS with or without E2 treatment for 24 h, intracellular lipid accumulation *in vitro* was measured by the ORO staining assay. Representative ORO staining **(AI)** and quantification **(AII)** of red dot intensity in THP-1 cells after treatment with dissolving medium, E2, rosuvastatin, or E2 + rosuvastatin in the presence of 10% HS. FBS (5%)-treated THP-1 cells were set as the negative control (*n* = 10∼20 cells/field/slice, *n* = 3 slices/group). **(B)** Eight-week-old ApoE^–/–^ mice were subjected to ovariectomy. Seven days later, they were randomized to three groups: high-fat diet (HFD) group (*n* = 8); HFD + E2 (0.25 mg/kg/day) (*n* = 8); HFD + rosuvastatin (5 mg/kg/day) (*n* = 8); HFD + E2 (0.25 mg/kg/day) + Rosuvastatin (5 mg/kg/day) (*n* = 8). Treatments continued for 20 weeks until euthanasia. Bodyweights of mice at time of euthanasia were shown in violin plots. **(C)** After euthanasia, peripheral blood was collected from the abdominal aorta. Violin plots show the distribution of serum concentration of alanine aminotransferase (ALT) **(CI)**, aspartate aminotransferase (AST) **(CII)**. **(D)** Violin plots show the distribution of serum concentration of TC **(DI)**, TG **(DII)**, HDL-C **(DIII)**, and LDL-C **(DIV)** of the four groups of mice (*n* = 8 mice/group). **(E)** Representative photomicrographs **(EI)** and quantification of plaques in the lumen **(EII)** of *en face* aortic arch and descending aorta of the four groups of mice (*n* = 8 mice/group). **(F)** Aortic roots were prepared as rapid freezing sections and subjected to H&E staining for detection of plaque area of the three groups of mice. Representative images of H&E staining **(FI)** of the aortic root and plaque area **(FII)** of the four groups of mice (*n* = 3 sections/mouse/group, *n* = 8 mice/group). **(G)** Liver tissue were prepared as rapid freezing section and subjected to ORO staining for detection of lipids accumulation. Representative images of liver ORO staining are shown **(GI)** (*n* = 3 sections/mouse/group, *n* = 8 mice/group). Densitometric quantification was conducted using Image J and values are expressed as the fold change compared with control and are presented as mean ± SEM (*n* = 8 mice/group). Student’s *t*-test was used to evaluate the significance in differences between two groups of observations, *P < 0.05; **P < 0.01; ***P < 0.001.

ESRα is a targeted receptor of E2, treatment with 10% HS enhanced the immunoreactivity of ESRα in THP-1 cells and HepG2 cells, in particular the mean fluorescence intensity in the nucleus was increased dramatically (Supplementary Figure S3), we postulated that E2/ESRα might regulates SREBP-1 expression. Based on the GEPIA database, we found that SREBP-1 expression was strongly correlated with ESRα expression in the liver, blood as well as in TCGA (The Cancer Genome Atlas) normal tissue (Supplementary Figure S4). We explored the pattern of SREBP-1-expression variation by manipulating ESRα expression. ESRα overexpression caused a significant increase in mRNA (Figure 3A) and protein expression of ESRα and n-SREBP-1 (Figure 3B). Correspondingly, knockdown of ESRα expression with shRNA resulted in downregulated mRNA and protein expression of ESRα and SREBP-1. The immunofluorescence analysis of SREBP-1 by manipulating ESRα expression in THP-1 and HepG2 were consistent with the WB results (Figure 3C). Moreover, to assess the role of SREBP-1 in E2/ESRα-mediated LDL uptake and lipid accumulation in foam cells, E2 + HS-treated HepG2 cells were overexpressed and/or depleted of ESRα/SREBP-1 expression. ESRα overexpression significantly attenuated lipid accumulation and LDL-uptake effects in HepG2 cells that had been stimulated by HS treatment; depletion of ESRα with shRNA restored those effects (Figures 3D,E). Silencing SREBP-1 expression also abolished the inhibition of lipid-accumulation effects of ESRα overexpression, which suggested that the E2/ESRα/SREBP-1 regulatory pathway was involved in the E2-related lipid-metabolism mechanism.

### ESRα Regulated SREBP-1 Activity Partly *via* the mTOR/p-mTOR Pathway in HepG2 Cells

**FIGURE 4:**
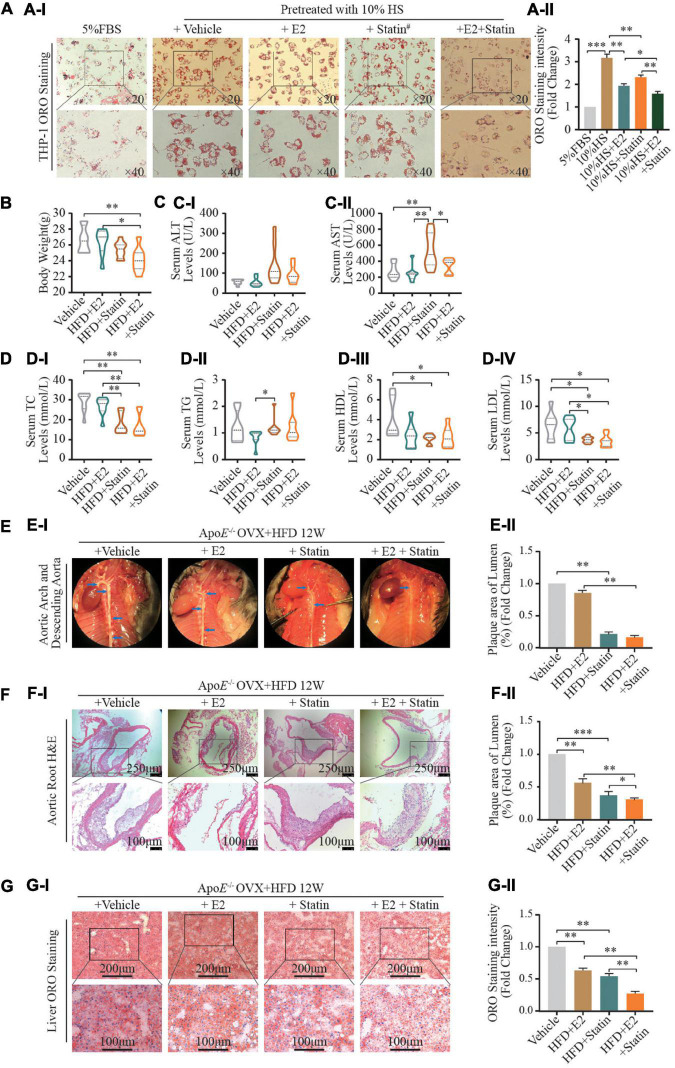
E2/ESRα regulates SREBP-1 expression. **(A–C)** HepG-2 cells were cultured for 7 days in phenol red-free medium supplemented with 5% FBS, cells were transfected with pcDNA control, ESRα, ESRα and ESRα shRNA, after gene transfection, the cells were subsequently cultured with 10 nM E2 for 48 h and harvested for RT-qPCR **(A)**, western blotting **(B),** and immunofluorescence **(C)** to detect the mRNA and protein expression alteration of ESRα, pre-SREBP-1 and n-SREBP-1. immunofluorescence was visualized under confocal microscopy; DAPI stains the nucleus (blue), ESRα (green) (*n* = 5∼8 cells/field/slice, *n* = 3 slices/group). **(D)** Representative ORO staining **(DI)** and quantification **(DII)** of red dot intensity in HepG2 cells after interference with pcDNA control, ESRα, ESRα & ESRα shRNA, and ESRα & SREBP-1 shRNA plasmid. **(E)** Representative images **(EI)** and quantification **(EII)** of intracellular LDL in HepG2 cells after interference with pcDNA control, ESRα, ESRα and ESRα shRNA, and ESRα and SREBP-1 shRNA plasmid. Red: DyLight 550-marked LDL. **(F,G)** After gene transfection, HepG2 and THP-1 cells were subsequently cultured with or without p-mTOR inhibitor AZD8055 (20 nM) for 48 h, cells were harvested for western blotting, representative blotting images **(FI,GI)** and quantification **(FII,GII)** of mTOR, p-mTOR, pre-SREBP-1 and n-SREBP-1, and ESRα. Data illustrated on the bar graph are the mean ± SD. All representative images presented were repeated in three independent experiments. Student’s *t*-test was used to evaluate the significance in differences between two groups of observations, *P < 0.05; **P < 0.01; ***P < 0.001.

Over recent years, several studies have revealed that mammalian target of rapamycin complex 1 (mTORC1) has a crucial role in promoting lipid biosynthesis by activating SREBP-1 (26, 27). To assess if E2/ESRα-activated SREBP-1 expression was mediated by mTORC1, we treated ESRα-overexpressed cells with a mTORC1 inhibitor (AZD8055) for 24 h. ESRα overexpression did not affect expression of mTOR or p-mTOR in two cell lines (Figures 4F,G). AZD8055 treatment decreased p-mTOR protein expression in HepG2 cells and THP-1 foam cells, and it decreased n-SREBP-1 expression in HepG2 cells (Figures 3F,G and Supplementary Figure S5), which suggested that ESRα regulated SREBP-1 activity (at least in part) *via* the mTOR/p-mTOR pathway in hepatocytes.

### E2 Enhanced Cellular Sensitivity to Statins *in vivo* and *in vitro*

Statins are competitive inhibitors of 3-hydroxy-3-methylglutaryl-coenzyme A reductase (HMGCR). Statins are a class of lipid-lowering medications that reduce illness and mortality in those who are at high risk of cardiovascular disease. We wished to assess the relationship between inhibition of HMGCR expression and E2 regulation in postmenopausal atherosclerotic disease caused by a combination of statins and E2. Hence, E2 (2 nM) or its vehicle alone, or combined with rosuvastatin (5 μM), were used to treat HS-stimulated THP-1 foam cells. Compared with the vehicle group, treatment with E2 and rosuvastatin decreased lipid accumulation in foam cells (Figure 4A). Noticeably, the combination of E2 with rosuvastatin dramatically reduced lipid accumulation in THP-1 foam cells when compared with that in the vehicle group or monotherapy group (*P* < 0.01).

We wished to investigate the effect on hepatic lipid disposition and formation of atherosclerotic plaques in arteries. *In vivo*, E2 (0.25 mg/kg/day) combined with rosuvastatin (5 mg/kg/day) or an equal volume of saline solution were used to treat normal diet-fed OVX ApoE^–/–^ mice. After 12 weeks of treatment, supplementation with E2 alone (0.25 mg/kg/day) did not affect bodyweight or lipid profiles (Figures 4B–D) in contrast with the HFD-fed saline-treated OVX group. Noticeably, therapy with rosuvastatin or a combination of E2 and rosuvastatin resulted in reduced bodyweight as well as decreased plasma levels of TC and LDL-C in HFD-fed OVX ApoE^–/–^ mice when compared with those in the HFD-fed saline-treated OVX group (Figures 4B–D). Rosuvastatin significantly increased serum levels of aspartate transaminase and alanine aminotransferase; E2 and rosuvastatin co-treatment restored these effects, which suggested that E2 relieved statin-caused hepatic damage (Figure 4C). Administration of E2 or rosuvastatin alleviated aortic-root plaque formation and relieved lipid deposition compared with that in saline-treated OVX controls (Figures 4E–G). Remarkably, combined treatment of E2 and rosuvastatin enhanced these cardioprotective effects. In contrast to the monotherapy group (E2 or rosuvastatin), combined treatment resulted in less fatty-liver damage and a smaller plaque size in the aortic root.

Taken together, these results implied that E2 enhanced cellular sensitivity to statins *in vivo* and *in vitro*. Combination of E2 and statins might have synergistic cardioprotective effects by regulating lipid metabolism.

## Discussion

The metabolic effects of E2 range from weight gain to the development of metabolic syndrome, and are associated with cardiovascular and hepatic manifestations. Studies on animal models of atherosclerosis have provided compelling evidence that physiological E2 levels potently attenuate the early and advanced stages of atherosclerotic lesions in females.

Atheroprotection by E2 in atherogenic transgenic mouse models with targeted inactivation of the LDL receptor (28) and ApoE (29, 30) has been demonstrated. In these models, E2 hindered atherosclerosis development. E2 deficiency induced by fulvestrant in female rats resulted in weight gain as well as disturbed metabolism of glucose and lipids (31). Consistent with those findings, we found that E2 supplementation attenuated lipid accumulation and LDL-uptake in cholesterol-loaded THP-1 macrophages (foam cells) and HepG2 cells. Moreover, E2 administration decreased fatty-liver damage and relieved plaque formation in the heart chambers of OVX Apo*E*^–/^*^–^* mice. Mennatallah et al. (31) reported that an OVX rat model was associated with dyslipidemia as demonstrated by significant increases in levels of TG, TC, and LDL-C as well as a significant decrease in the HDL-C level. In our study, the LDL-C level was negatively correlated with the serum E2 level in postmenopausal women but, in the OVX mouse model, inhibition of atherosclerotic lesions using exogenous E2 (supplementation for 12 and 20 weeks) did not influence serum lipid profiles or bodyweight. A likely possibility is that continuous therapy in a mouse model was different with circulating E2 levels in humans; physiological rhythmic release of hormones may have a more comprehensive impact upon metabolism kinetics.

In general, E2 exerts its physiological effects through ESRα and ESRβ. Functional ESRs are expressed in macrophages (32), vascular endothelial cells (33), and smooth muscle cells (34). Expression of ESRs has been reported to be downregulated in the atherosclerotic vessels of postmenopausal women (35). In a combined ApoE/ERα deletion model, ERα was reported to be a major mediator of E2 protection in advanced atherosclerotic lesions (36), and ERα deficiency abolished the atheroprotection effect of E2 in ApoE ^–/–^ mice (36). However, the relative contribution of E2 signaling through ESRα with regard to the metabolism of lipids and lipoprotein and ASCVD is not known.

SREBP-1 is a transcription factor expressed ubiquitously. It is an adipocyte-differentiation factor which directly regulates the transcription of > 200 genes involved in the *de novo* synthesis of fatty acids, TG and cholesterol. The phenotypes of published germline and tissue-specific knockout/transgenic mice for the SREBP pathway have revealed the potential of SREBP-1 to reduce fatty-acid synthesis, enhance insulin secretion, and alter the inflammatory response in the liver, intestine, pancreas, and immune system. Previously, we discovered that decreased SREBP-1 expression was a risk factor for coronary artery disease (CAD), and that expression of SREBP-1 mRNA in carotid plaques correlated with the corresponding value in circulating leukocytes in CAD patients undergoing carotid endarterectomy (19). In the present study, after stratification by various clinical parameters, we found SREBP-1 expression to correlate with the serum E2 level. Expression of ESRα protein strongly correlated with expression of SREBP-1 protein in the liver, blood and TCGA normal tissue based on the GEPIA database; moreover, manipulating ESRα expression simultaneously led to alteration of n-SREBP-1 expression. Pedram et al. showed that activation of membrane-localized ESRα by the E2 agonist prolyl-pyrazole-triol could induce adenosine monophosphate-activated protein kinase to phosphorylate SREBP-1 in the liver, thereby preventing its proteolytic cleavage by site-1 protease (37). Consequently, SREBP-1 was sequestered in the cytoplasm, thereby preventing expression of cholesterol synthesis-associated genes (37). In the present study, under standard culture conditions, SREBP-1 was localized to the nucleus in HePG2 cells, and hyperlipidemic or E2-hyperlipidemic co-treatment significantly increased the ratio of cytoplasm expression:nucleus expression of SREBP-1. As a critical lipid metabolism-related transcription factor, nuclear SREBP-1 regulates expression of target genes. Retention of cytoplasmic SREBP-1 might lead to repressed expression of cholesterol- and lipid synthesis-related genes that result in decreased accumulation of intracellular lipids in the liver. Interestingly, the pattern of SREBP-1 expression in THP-1 macrophages was different from that in HepG2 cells. Barely expressed under normal conditions, hypercholesterolemia stimulation dramatically increased its nuclear expression, whereas E2 and hyperlipidemic co-treatment increased its cytoplasmic retention. These data suggested that E2 might work as a “fine tuning” regulator to maintain lipid homeostasis during foam-cell formation. Therefore, we speculated that E2/ESRα might regulate lipid homeostasis directly/indirectly *via* SREBP-1 signaling. Subsequently, we globally identified the promoters occupied by ESRα in human hepatocyte lines (HepG2, LO2, LM3) using chromatin immunoprecipitation sequencing, but, the ESRα-binding DNA sequence was exclusive of the SREBP-1 promoter region. AKT/mTOR has been reported to be an upstream SREBP-1 regulating pathway which can modulate hepatic lipid kinetics by affecting COPII-dependent SREBP-1 processing (38), and mTOR is an essential regulator of lipogenic metabolism because it activates SREBP-1 cleavage (39). In the present study, an inhibitor of the AKT/mTOR pathway, AZD8055, partly abolished ESRα-induced SREBP-1 expression in HepG2 cells, which implied that SREBP-1 activation by ESRα could be related to the mTOR pathway. However, inhibition of p-mTOR did not affect SREBP-1 expression in THP-1 foam cells. The reciprocal regulation between ESRα and SREBP-1 is complex, and further research is needed to understand the regulatory mechanism between these two factors.

Our previous case–control study uncovered that downregulated SREBP-1 expression in peripheral leukocytes was an independent risk factor for CAD. However, those effects seem to contradict with the data that treatment with 10% HS upregulated SREBP-1 expression and increased lipid accumulation in foam cells. Overexpression of SREBP-1c protein has been reported to lead to disturbances in the metabolism of carbohydrates and lipids, and hepatic lipid content (40). In OVX rats, enhanced hepatic expression of SREBP-1c may be a cause of excessive hepatic lipid production through subsequent activation of fatty acid synthase (FASN). Whether an increase in SREBP-1 expression is a protective factor or a risk factor for CAD is influenced by two factors. On the one hand, estrogen-induced SREBP-1 expression regulates lipid metabolism with the aim of restoring lipid homeostasis. On the other hand, abnormal alterations in lipid levels can feedback and adjust SREBP-1 expression. Therefore, upregulated expression of SREBP-1 seems to related to irregular lipid profiles. The interactive mechanism between SREBP-1 expression and lipid metabolism merits further exploration.

*SREBP-1* can produce two proteins, SREBP-1a and SREBP-1c, which are derived from different promoters (41). In humans, SREBP-1c primarily regulates fatty-acid metabolism, such as *FASN* (42, 43). SREBP-1a, with 1147 amino acids, is 47% identical to SREBP-2 in humans. The transcriptional activity of SREBP-2 corresponds to SREBP-1a on account of the almost identical length of the N-terminal domain. SREBP-2 is mainly responsible for cholesterol-related genes, such as *HMGCR* (a rate-limiting enzyme in cholesterol synthesis) and the low-density lipoprotein receptor gene (44). SREBP-1a targets both sides of genes. However, there are overlapping functions between individual SREBPs.

Statins are the most commonly used class of drugs to treat and prevent CAD worldwide. Statins strongly induce HMGCR *via* a SREBP-2 pathway (45). If statins bind to HMGCR, they block access of the natural substrate HMG-CoA to the catalytic site and thereby interfere with cholesterol synthesis. On the basis that E2 regulated SREBP-1 expression, we speculated that E2 might influence the cellular sensitivity of statins by cross-regulating SREBPs. E2 and statin co-treatment significantly decreased lipid accumulation *in vitro*, after HFD consumption for 12 weeks, administration of E2 and a statin together significantly relieved blood-lipid disorders and hindered progression of atherosclerosis and fatty-liver damage in OVX ApoE^–/–^ mice. This observation has an important translational implication because clinical studies exploring E2 supplementation as atheroprotection agents have been hindered by the increased risk of complications of high-dose E2 treatment in postmenopausal women. Investigation of the utility of low-dose, E2-based combination treatment against CAD is needed.

Amparo and colleagues also discovered that E2 inhibits and delays the development of early lesions *via* ERα-independent mechanisms because they found no significant interaction between the ERα genotype and hormone treatment or hormone status (46). The differences between our findings and their observations might be because the pleiotropic effects of hormone therapy on the vascular system and cells might differ depending not only on hormone status, but also on the atherosclerosis stage in the underlying physiological condition. We need to know to what extent and how the cessation of endogenous E2 production affect ESR expression/function in the regulatory network of lipid kinetics and thereby enhances/attenuates their pro- or anti-atherogenic effects.

## Conclusion

The present study aids deeper understanding of the ESRα/SREBP-1-dependent mechanisms of E2 linked to cholesterol metabolism in postmenopausal women. We also provide evidence that E2 enhances statin sensitivity which might have synergistic anti-atherosclerotic effects in the early stages of cardiovascular lesions and mild hepatic injury. This knowledge is potentially important because deeper understanding of this system may lead to stronger comprehension of how to optimize hormone therapies for protection against cardiovascular disease in postmenopausal women.

## Data Availability Statement

The original contributions presented in this study are included in the article/Supplementary Material, further inquiries can be directed to the corresponding author/s.

## Ethics Statement

The studies involving human participants were reviewed and approved by Medical Ethics Committee of Taihe Hospital of the Hubei University of Medicine. The patients/participants provided their written informed consent to participate in this study. The animal study was reviewed and approved by Animal Care and Use Committee of Hubei University of Medicine.

## Author Contributions

CP: concept, funding, and critical review. FX and CP: design and writing. XLi, YX, and DC: materials. XX, XLv, and GY: data collection and/or processing. FX and CP: analysis and/or interpretation. FX: literature search. All authors contributed to the article and approved the submitted version.

## Conflict of Interest

The authors declare that the research was conducted in the absence of any commercial or financial relationships that could be construed as a potential conflict of interest.

## Publisher’s Note

All claims expressed in this article are solely those of the authors and do not necessarily represent those of their affiliated organizations, or those of the publisher, the editors and the reviewers. Any product that may be evaluated in this article, or claim that may be made by its manufacturer, is not guaranteed or endorsed by the publisher.
