# Supplementary material for: Estrogen Mediates an Atherosclerotic-Protective Action via Estrogen Receptor Alpha/SREBP-1 Signaling
Source: Front Cardiovasc Med. 2022 Jul 5;9:895916. doi: 10.3389/fcvm.2022.895916 (PMC9294214; doi:10.3389/fcvm.2022.895916)
Supplement: Supplementary file 1 [file Data_Sheet_1.DOCX]

**Methods:**

**S1.1 Participants**

We enrolled female participants who experienced natural menstrual states, based on the following criteria: (1) providing vital data, such as age, sex, sex hormone levels, and serum lipid profiles; (2) no presence of complications or conditions that affect menopausal status or lipid metabolism, such as uterectomy, bilateral ovariectomy, premature ovarian failure, history of irregular menstruation during premenopause, pregnancy, lactation, psychiatric illness, acute cardiovascular or cerebrovascular disease, chronic respiratory disease, familial hypercholesterolemia, malignant tumors, hepatic or renal dysfunctions; (3) no use of medications that affect the natural state of menopause or lipid metabolism, including estrogen, progesterones, androgens, glucocorticoids, statins, fibrates, thyroid hormones, anti-thyroid drugs, nonsteroidal anti-inflammatory drugs, β-adrenoceptor blockers, or thiazide diuretics, over the preceding three months. Determination of menopausal status was based on the responses to questions regarding menstrual irregularity and amenorrhea in the self-reported questionnaire. “Perimenopause” was defined as the presence of menses within the previous 3 months with a reduction in cycle predictability in the year preceding examination, or 3–11 months of amenorrhea. As a result, 80 pre-menopausal, 20 peri-menopausal and 131 post-menopausal female subjects were eligible for investigation in this study.

**S1.2 Analysis of mRNA expression by qRT-PCR**

Total RNA was extracted from cells using Trizol reagent (Invitrogen, CA, USA) according to standard procedures. The quality and integrity of the extracted RNA were assessed using NanoDrop ND-1000 spectrophotometer and ethidum bromide/agarose gel electrophoresis. The RNA was stored at 80 ℃ until used for quantitative-real time PCR (qRT-PCR) analysis. One mg of total RNA was reverse transcribed into single-strand cDNA using first strand cDNA synthesis kit (Takara, Dalian, China) and the mRNA expression was quantified by qRT-PCR. The relative gene expression was normalized to housekeeping gene GAPDH and calculated using the formula 2^–△△^CT. The sequences of gene-specific primers are listed in Table S1.

**S1.3 Western blot**

Cells or tissues were lysed in radio-immunoprecipitation assay (RIPA) lysis buffer containing a halt protease and phosphatase inhibitor cocktail. Total lysates were then centrifuged to remove debris and protein concentration was measured by BCA assay. Equal amounts (30-50μg) of lysates were separated in sodium dodecyl sulfate polyacrylamide gel (7.5-15%) by electrophoresis, transferred to PVDF membranes, and blocked with 5% skim milk for reducing non-specific binding. Membranes were then incubated with the primary antibodies against SREBP-1，SREBP-2, ESRα (Abcam, Cambridge, UK), mTOR, PmTOR, PI3K (Cell signaling Technology, Danvers, MA), GAPDH（Proteintech, Wuhan, China）overnight at 4 ℃ followed by further incubation with an appropriate HRP-conjugated secondary antibody for 1h at room temperature. An enhanced chemiluminescence (ECL) detection system was used to detect immunocomplexes and images were acquired using the the ChemiDoc™ Touch imaging system (Bio-Rad Laboratories, Hercules, CA, USA).

**S1.4 LDL-C uptake assay**

Then LDL-C uptake was determined according to the manufacturer’s protocol (Cat#133127, Abcam, Cambridge, UK). Briefly, 4,000 HepG2 Cells and THP-1 macrophages were plated in 96-well plates and cultured in medium supplemented with 10nM E2 or its vehicle in the presence of 10% FBS for 24h. After treatment, culture medium was aspirated and replaced with LDL-DyLight 550 working solution with 10nM E2 or its vehicle. Cells were then incubated for 3 hours at 37 ℃, followed by three washes with PBS, and then visualized via fluorescent microscopy with excitation and emission wavelengths of 540 and 570 nm, respectively. After visualization, cells were fixed with a cell-based fixative solution for 10 min. Cells were then washed with tris-buffered saline plus 0.1% Triton X-100 for 5 min, followed by 30-min blocking with Cell-Based Assay Blocking Solution. Cells were then stained with rabbit anti-LDL receptor primary antibody and Dylight 488-conjugated secondary antibody. Images were taken with a fluorescent microscope with excitation and emission wavelengths of 485 and 550 nm, respectively.

**S1.5 Imunofluorescence staining of Cells**

Cells were seeded into a 6-well plate. At the next day they were incubated with different treatment for 24h. Then these cells underwent fluorescent staining according to published methods. In brief, cells were fixed in 4% paraformaldehyde (PFA) for 30 mins, washed three times in PBs, permeabilized in 0.3% Trition X-100 on ice for 5 min, blocked in 5% FBS for 30 mins, incubated with primary antibody against ESRα , SREBP-1, SREBP-2 () at 4℃ overnight，and then incubated with FITC-conjugated secondary antibody (Invitrogen, #185182, 1:1000) for 1h. Nuclei were stained with DAPI (CAT, #, 1:1000). Immunofluorescence images were visualized and photographed by using a laser scanning confocal microscopy (FV3000RS; Olympus Corporation, Tokyo, Japan).

**S1.6 Murine surgical model of menopause and atherogenesis**

Animal experiments followed the *Guide for the Care and Use of Laboratory Animals* (National Institutes of Health, Bethesda, MD, USA) and their conduct was approved by the Animal Care and Use Committee of Hubei University of Medicine. Five-week-old *ApoE ^-/-^* female mice were purchased from Biocytogen (Nanjing, China). Mice were lived in a temperature-controlled space, keeping a 12-h light/dark cycle. After three weeks of acclimatization were randomized to bilateral Ovariectomized (OVX) or a surgical sham operation. Surgeries were performed on anesthetized female mice (1% pentobarbitone). Briefly, the dorsum of each mouse was incised and both ovaries were removed. The wound site was monitored for bleeding, muscle and skin layers were closed with sterile synthetic absorbable sutures. The site was then treated with povidone-iodine, and mice were allowed to recover in a clean cage on a heating pad and move to SPF Animal Center in Hubei University of Medicine. One week after surgery, mice were stratified into three groups of five in the first stage and stratified into four groups of eight in the second stage: the diagram of two parts are shown below.

Part 1


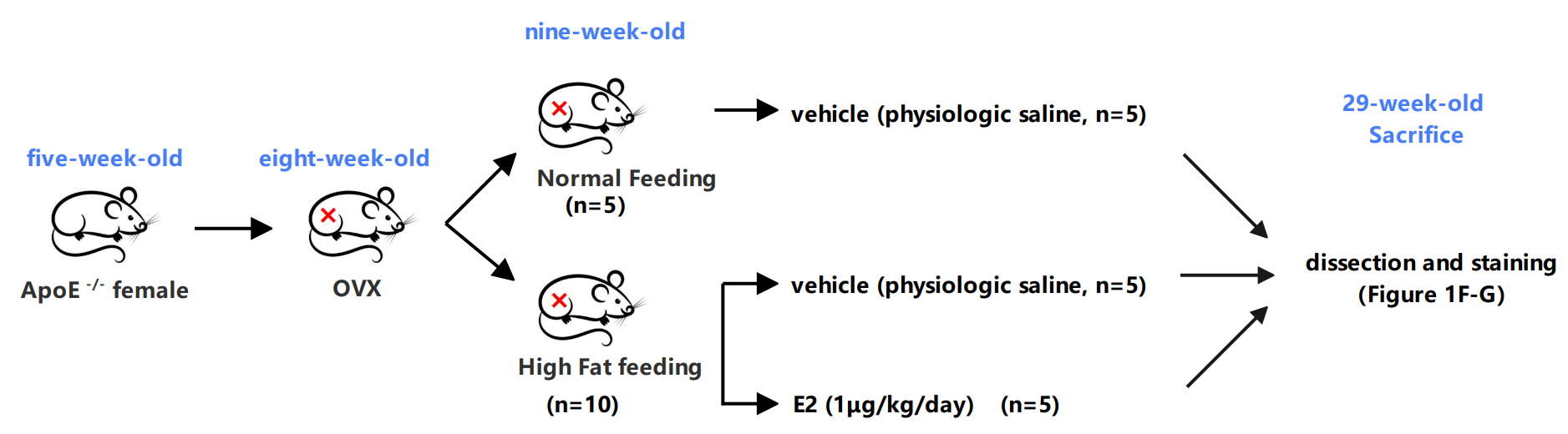


Part 2


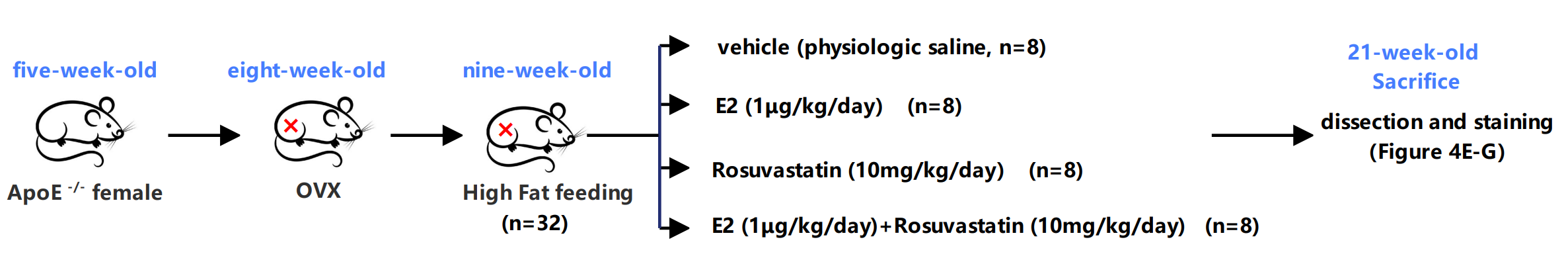


Figure S1 Diagram of the animal research

**Table S1 Biochemical and hormone levels of mixed hyperlipidemia serum***

| **Characteristic** | **Concentration** |
| --- | --- |
| TC (mmol/L) | 6.42 |
| TG (mmol/L) | 2.15 |
| HDL-C (mmol/L) | 1.42 |
| LDL-C (mmol/L) | 4.37 |
| Apo-A1 (g/L) | 1.85 |
| Apo-B (g/L) | 1.12 |
| Glucose (mmol/L) | 6.21 |
| LH (IU/L) | 24.15 |
| FSH **(**IU/L**)** | 81.84 |
| Estradiol (pmol/L) | 77.32 |
| Prolactin (ug/L) | 6.65 |

***** Blood sample were obtained from 26 post-menopause female participants who suffered from hyperlipidemia, 5ml serum of each sample were mixed together, which used as mixed hyperlipidemic serum (HS) to treat cells.

**Table S2 Primer sequences used for qPCR**

| **Gene name** | **Primer sequence 5’-3’** | **Method** |
| --- | --- | --- |
| **SREBP-1** | F: TGCATTTTCTGACACGCTTC  R: CCAAGCTGTACAGGCTCTCC | qPCR |
| **SREBP-2** | F: TGGCTTCTCTCCCTACTCCA  R: GAGAGGCACAGGAAGGTGAG | qPCR |
| **GAPDH** | F: GAAGGTGAAGGTCGGAGTC  R: GAAGATGGTGATGGGATTTC | qPCR |

**Table S3 shRNA Sequence lists**

| **Gene name** | **TARGET SEQUENCE LOOP SEQUENCE GUIDE SEQUENCE** |
| --- | --- |
| **hSREBP1[shRNA#1]** | TATTCCGGGAACATCTCTTAGCTCGAGCTAAGAGATGTTCCCGGAATA |
| **hSREBP1[shRNA#2]** | GTGACTTCCCTGGCCTATTTGCTCGAGCAAATAGGCCAGGGAAGTCAC |
| **hSREBP1[shRNA#3]** | TGAGGCTCCTGTGCTACTTTGCTCGAGCAAAGTAGCACAGGAGCCTCA |
| **hESRα[shRNA#1]** | GTGTGCCTCAAATCTATTATTCTCGAGAATAATAGATTTGAGGCACAC |
| **hESRα[shRNA#2]** | CTACAGGCCAAATTCAGATAACTCGAGTTATCTGAATTTGGCCTGTAG |
| **hESRα[shRNA#3]** | ATGCTTCAGGCTACCATTATGCTCGAGCATAATGGTAGCCTGAAGCAT |

**Table S4. Baseline and clinical characteristics of the participants.**

| **Characteristic** | **Pre-menopause(n=80)** | **Peri-menopause(n=20)** | **Post-**  **menopause(n=131)** | ***P^1^*** | ***P^2^*** | ***P^3^*** |
| --- | --- | --- | --- | --- | --- | --- |
|  |  |  |  | ***Pre-***  ***vs***  ***Peri-*** | ***Pre-***  ***vs***  ***Post-*** | ***Peri-***  ***Vs***  ***Post-*** |
| **Demographics** |  |  |  |  |  |  |
| Age (years) **^*^** | 43.63±9.21 | 50.6±4.39 | 60.18±4.26 |  |  |  |
| **Clinical parameters** | | | | | | |
| TC (mmol/L) | 4.28±0.93 | 4.68±1.22 | 4.39±1.19 | 0.118 | 0.478 | 0.329 |
| TG (mmol/L) | 1.38±0.95 | 1.22±0.76 | 1.35±0.76 | 0.472 | 0.800 | 0.463 |
| HDL-C (mmol/L) | 1.21±0.37 | 1.39±0.37 | 1.30±0.41 | ***0.047*** | 0.107 | 0.325 |
| LDL-C (mmol/L) | 2.09±0.56 | 2.20±0.61 | 2.16±0.67 | 0.428 | 0.471 | 0.761 |
| Apo-A1 (g/L) | 1.51±0.23 | 1.65±0.22 | 1.57±0.28 | ***0.016*** | 0.106 | 0.221 |
| Apo-B (g/L) | 0.86±0.18 | 0.89±0.18 | 0.86±0.20 | 0.641 | 0.761 | 0.531 |
| LH (IU/L) **^△^** | 4.29(2.98,5.97) | 25.17(10.70,40.04) | 12.37(5.36,25.89) | ***<0.001*** | ***<0.001*** | ***0.019*** |
| FSH **(**IU/L**)** | 6.07(4.32,7.67) | 63.61(27.58,100.99) | 28.86(9.39,72.06) | ***<0.001*** | ***<0.001*** | ***0.037*** |
| Estradiol (pmol/L) | 469.81±400.04 | 242.33±159.53 | 59.28±24.77 | ***<0.001*** | ***<0.001*** | ***<0.001*** |
| Prolactin (ug/L) | 9.84(6.18,14.27) | 8.91(6.84,14.10) | 8.32(5.71,11.52) | 0.612 | 0.494 | 0.485 |

**^*^**normally distributed data were illustrated in the form as mean±SD，and their comparison between groups are using *student t-text*. **^△^**non-normal distributed data were illustrated in the form of IQR, and their comparison between groups are using *non-parameter analysis*.

**Table S5** **Pearson/Spearman correlation coefficients between** **blood lipids levels and E2 levels**

|  | **E2** | | | | | | | | | | |
| --- | --- | --- | --- | --- | --- | --- | --- | --- | --- | --- | --- |
|  | **Pre-menopause(n=80)** | |  | **Peri-menopause(n=20)** | |  | **Post-**  **menopause(n=131)** | |  | **Overall (n=231)** | |
|  | **r** | ***P*** |  | **r** | ***P*** |  | **r** | ***P*** |  | **r** | ***P*** |
| **TC^*^** | -0.036 | 0.754 |  | 0.131 | 0.583 |  | ***-0.240*** | ***0.006*** |  | -0.109 | 0.097 |
| **TG** | -0.109 | 0.334 |  | -0.005 | 0.983 |  | -0.023 | 0.794 |  | -0.052 | 0.430 |
| **LDL-C** | -0.210 | 0.061 |  | 0.053 | 0.826 |  | ***-0.239*** | ***0.006*** |  | ***-0.134*** | ***0.042*** |
| **HDL-C** | ***0.236*** | ***0.035*** |  | 0.208 | 0.379 |  | 0.241 | 0.006 |  | -0.120 | 0.069 |
| **Apo-A1** | 0.123 | 0.275 |  | 0.218 | 0.357 |  | -0.143 | 0.104 |  | -0.102 | 0.121 |
| **Apo-B** | ***-0.262*** | ***0.019*** |  | 0.050 | 0.834 |  | -0.149 | 0.090 |  | -0.036 | 0.584 |
| **SREBP-1^△^** | 0.083 | 0.465 |  | 0.317 | 0.173 |  | ***0.279*** | ***0.001*** |  | ***0.353*** | ***< 0.001*** |

**^*^** Pearson correlation coefficients were used to analyzed the correlation between TC, TG, LDL-L, HDL-C, Apo-A1, Apo-B and E2, **^△^** Spearman correlation coefficients were used to analyzed the correlation between SREBP-1 expression and E2.

**
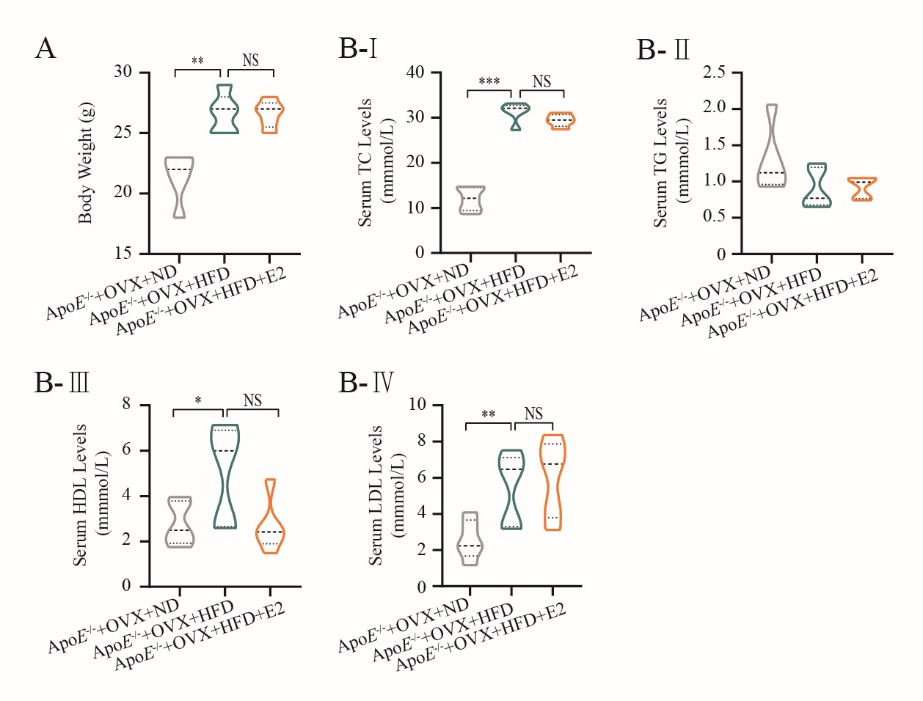
**

**Fig S1** **(A)** Eight-week-old ApoE−/− mice were subjected to ovariectomy. Seven days later, they were randomized to three groups: normal diet (NF) group (n = 5), high-fat diet (HFD) group (n = 5), or HFD + E2 (1 μg/kg/day) administration group (n = 5). Treatments continued for 20 weeks until euthanasia. Bodyweights of mice at time of euthanasia were shown in violin plots. **(B)** After euthanasia, peripheral blood was collected from the abdominal aorta. Violin plots show the distribution of serum concentration of TC (G-Ⅰ), TG (G-Ⅱ), HDL-C (G-Ⅲ) and LDL-C (G-Ⅳ) of the three groups of mice (n = 5 mice/group).


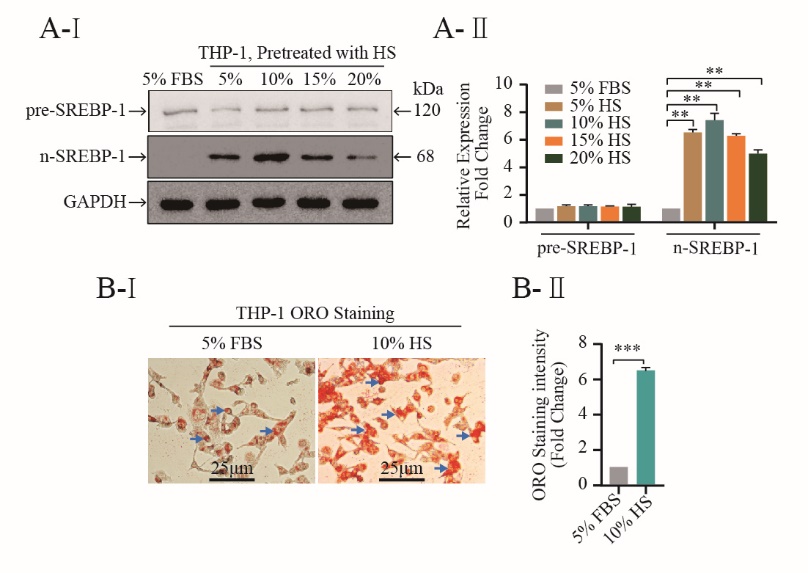


**Fig S2 (A)** THP-1 cells were treated with different concentrations HS for 24 hours. Total protein lysates were prepared and used for the measurement of precursor (pre-SREBP-1) and nuclear SREBP-1 (n-SREBP-1) expression by Western blot analysis. Representative images from at least three independent experiments are presented (A-I). GAPDH was served as a loading control. Relative band intensities of the target proteins compared to the loading control were quantified by densitometric analysis and are presented as bar diagrams shown in the corresponding panel (A-II). **(B)** THP-1 macrophages stimulated by 5% FBS and 10% HS for 24 hours. intracellular lipid accumulation *in vitro* was measured by the ORO staining assay. Representative images from at least three independent experiments are presented (B-Ⅰ). Total area of lipid droplets normalized to cell number (10 cells per each field) was determined using Image J software and shown in (B–Ⅱ). All representative images presented were repeated in three independent experiments. Data illustrated on the bar graph are the mean ± SD. Student’s t-test was used to evaluate the significance in differences between two groups of observations, * *P* < 0.05; ** *P* < 0.01; *** *P* < 0.001.


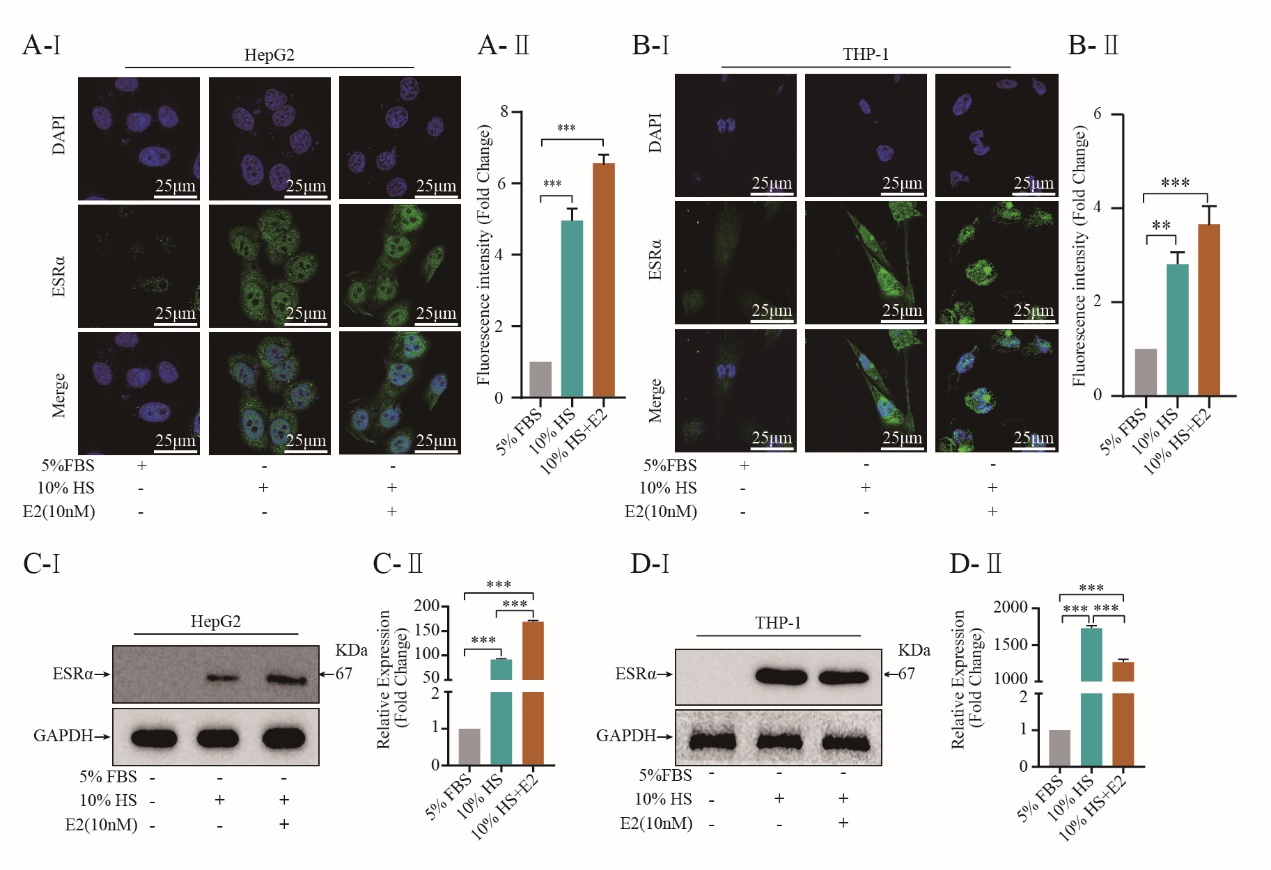


**Figure S3 Estrogen influences ESRα expression.** **(A, B)** HepG2 cells (A) and THP-1 cells (B) pretreated with or without 10% HS for 24 h in the presence or absence of E2 (10 nM). ESRα immunofluorescence were visualized under confocal microscopy; DAPI stains the nucleus (blue), ESRα (green) (n =5~8 cells/field/slice, n = 3 slices/ group). ESRα protein levels were further analyzed by western blot **(C, D)**. All representative images presented were repeated in three independent experiments. Data illustrated on the bar graph are the mean ± SD. Student’s t-test was used to evaluate the significance in differences between two groups of observations, * *P* < 0.05; ** *P* < 0.01; *** *P* < 0.001.


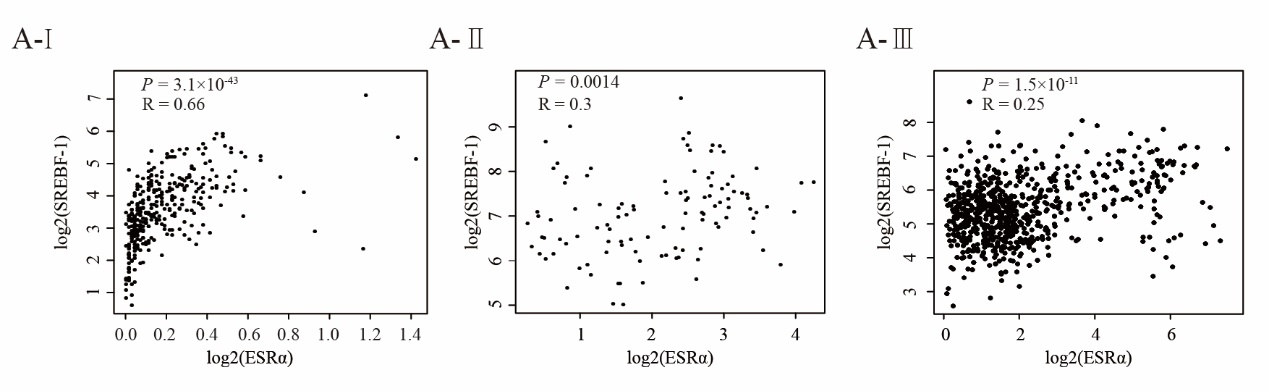


**Figure S4 E2/ESRα regulates SREBP-1 expression. (A)** Correlation of SREBP-1 expression and ESRα expression in the liver (A-I), blood (A-II) as well as in TCGA normal tissue (A-III). Data were obtained and analyzed by exploring the GEPIA database (<http://gepia.cancer-pku.cn/>). R represents Spearmen's correlation.


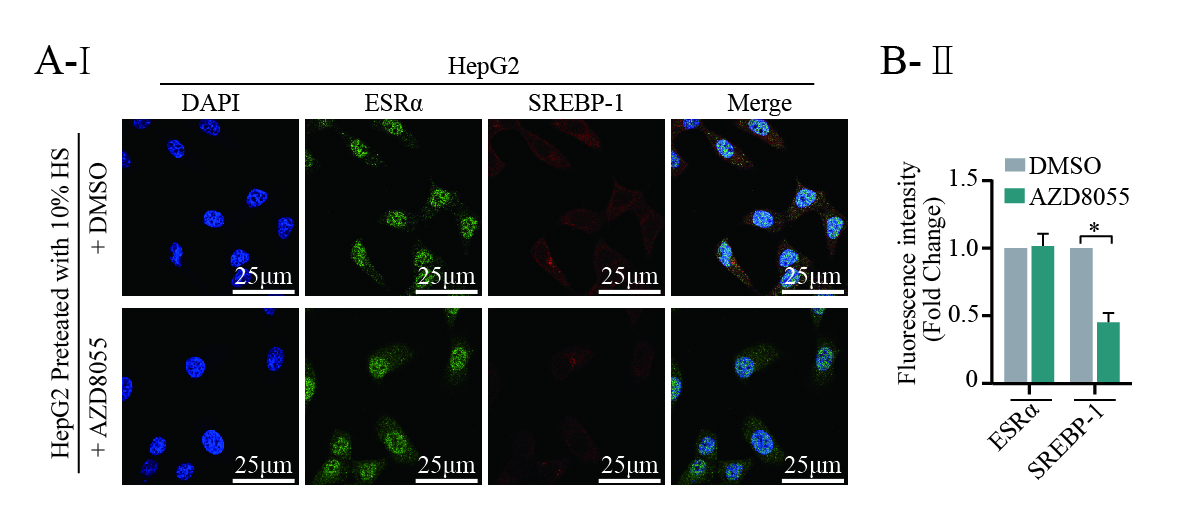


**Figure S5 E2/ESRα regulates expression of SREBP-1 partly *via* the mTOR/p-mTOR pathway.** HepG-2 and THP-1 cells were cultured for 7 days in phenol red-free medium supplemented with 10% HS, cells were transfected with pcDNA control (1ug/mL), ESRα (1ug/mL), after gene transfection, the cells were subsequently cultured with or without p-mTOR inhibitor AZD8055 (20 nM) for 48h, cells were harvested for immunofluorescence to detect the protein expression alteration of mTOR, p-mTOR, pre-SREBP-1 and n-SREBP-1 and ESRα. Immunofluorescence was visualized under confocal microscopy; Densitometric quantification was conducted using Image J and values are expressed as the fold change compared with control and are presented as mean ± SEM, (n=5~8 cells/field/slice, n = 3 slices/group). All representative images presented were repeated in three independent experiments. Data illustrated on the bar graph are the mean ± SD. Student’s t-test was used to evaluate the significance in differences between two groups of observations, **P* < 0.05; ***P* < 0.01; ****P* < 0.001.
